# Optimal NPK Fertilizer Combination Increases *Panax ginseng* Yield and Quality and Affects Diversity and Structure of Rhizosphere Fungal Communities

**DOI:** 10.3389/fmicb.2022.919434

**Published:** 2022-06-21

**Authors:** Jin Sun, Haoming Luo, Qian Yu, Baixin Kou, Yuxin Jiang, Lili Weng, Chunping Xiao

**Affiliations:** School of Pharmaceutical Sciences, Changchun University of Chinese Medicine, Changchun, China

**Keywords:** *Panax ginseng*, NPK fertilizers, fungal community structure, high-throughput sequencing, orthogonal experimental design, rhizome biomass, ginsenosides

## Abstract

Soil microorganisms affect crop rhizospheres *via* the transformation and transport of nutrients, which has important influences on soil fertility, carbon sequestration, and plant yield and health in agroecosystems. There are few reports on the effects of fertilizer application on the growth of *Panax ginseng* (C. A. Mey.) or the structure of its rhizosphere microbial communities. In this study, an orthogonal experimental design was used to explore the effects of nine different combinations of nitrogen (N), phosphorus (P), and potassium (K) fertilizers with different amounts and proportions on ginseng growth and accumulation of ginsenosides and the structure of rhizosphere soil fungal communities. Soil without fertilization was the control. With the combined application of NPK, ginseng growth and development increased. The fertilization scheme N_3_P_1_K_3_, with N fertilizer at 50 g·m^−2^, P fertilizer at 15 g·m^−2^, and K fertilizer at 60 g·m^−2^, had the most comprehensive benefit and significantly increased ginseng rhizome biomass and ginsenoside contents (Rg1, Re, Rf, Rg2, Rb1, Ro, Rc, Rb2, Rb3, and Rd). Amplicon sequencing showed that NPK application increased the diversity of fungal communities in ginseng rhizospheres, whereas richness was bidirectionally regulated by proportions and amounts of NPK. Ascomycota was the dominant fungal phylum in ginseng rhizosphere soil, and relative abundances decreased with combined NPK application. Combined NPK application increased the relative abundance of potential beneficial fungi, such as *Mortierella*, but decreased that of potentially pathogenic fungi, such as *Fusarium*. Correlation analysis showed that potential beneficial fungi were significantly positively correlated with ginseng rhizome yield and ginsenoside contents, whereas the opposite relation was observed with potential pathogenic fungi. Thus, in addition to directly increasing crop growth, precise NPK application can also increase crop adaptability to the environment by shaping specific microbial communities. The results of this study suggest that the combined effects of biotic and abiotic processes on agricultural production determine crop yield and quality.

## Introduction

*Panax ginseng* (C. A. Mey.) is a perennial herbaceous plant in the family *Araliaceae* that is primarily distributed in China, South Korea, and Russia (Abid et al., 2021). Ginseng is traditional and precious medicine in East Asia and has important medicinal and economic value (Li et al., 2022). The main active compounds in ginseng include ginsenosides, polysaccharides, and polypeptides, among others (Ratan et al., 2020). Ginsenosides are important saponins, and it is precise because of those compounds that ginseng is highly valued (Qiao et al., 2022). The quality of ginseng medicinal materials is closely related to ginsenoside monomer and total ginsenoside contents in rhizomes. To date, more than 30 ginsenosides have been reported in roots alone (Abid et al., 2021). Ginsenosides have a variety of pharmacological activities, including preventing neurological diseases, increasing immune function, delaying aging, and improving sleep (Kim et al., 2018; Jin et al., 2021). Although the importance and yield of ginsenosides have been widely studied, the yield of ginsenosides remains low (Zhang et al., 2021a; He et al., 2022). Therefore, it is urgent to optimize ginseng cultivation techniques and increase ginsenoside yields to meet medicinal needs.

*Panax ginseng* has a long cultivation history, and China has the largest cultivated area worldwide. The main area of ginseng production in China is Changbai Mountain in eastern Jilin Province (Xiao et al., 2016). With the rapid development of the ginseng industry, the ginseng cultivation area has increased significantly. The biosynthesis of active compounds in ginseng is affected by the physical, chemical, and microbial properties of soil (Chung et al., 2017). Fertilization is a universal practice to effectively increase soil nutrient supplies (Li et al., 2021a). However, prolonged, excessive, and unscientific fertilization methods have led to a series of ecological problems. The problems include soil pollution and destruction of microbial community structure, especially that of communities involved in nitrogen (N), phosphorus (P), and carbon (C) cycles, which lead to declines in crop growth and yield (Pavlidis et al., 2020; Shen et al., 2022). Fertilization also greatly affects microbial populations associated with potassium (K) and iron (Fe) cycles (Bose et al., 2014). Nitrogen, P, and K are essential elements for plant growth (Bernstein et al., 2019), and precise NPK application can significantly affect crop growth and metabolite accumulation (Sun et al., 2022). However, different crop species require different amounts and proportions of NPK, and the level of each element can affect plant growth and development (Bai et al., 2022). Therefore, the exploration and development of precise NPK fertilization schemes are prerequisites to positively affect soil health and improve crop yield and quality.

Soil microorganisms are the main engines of agroecosystems because they promote nutrient turnover in soils (Nie et al., 2018). In particular, fungi provide favorable conditions for crop growth and development by transforming and transporting nutrients in fertilizers and also by increasing nutrient acquisition from decomposing soil organic matter (Miao et al., 2022; Semenov et al., 2022). Therefore, the abundance and diversity of rhizosphere fungi have important effects on crop yield and quality (Gao et al., 2019). The combined application of NPK changes the structure of fungal communities in crop rhizosphere soil and provides a favorable environment for crop growth and development by increasing the abundance of potential beneficial fungi and decreasing that of potentially pathogenic fungi (Cai et al., 2021). Therefore, an in-depth understanding of how NPK fertilization affects soil microbial communities is essential to achieve a balance between agricultural development and ecological protection.

Although ginseng is planted widely and has important medicinal and economic value, understanding of ginseng rhizosphere soil microbial communities is limited. There remains a lack of systematic understanding of the effects of NPK fertilization on ginseng yield and quality and rhizosphere soil microbial communities. Therefore, in this study, an orthogonal experimental design was used to explore the effects of nine combinations of NPK fertilizers on ginseng growth indexes (plant height, stem, and leaf fresh weight, and rhizome fresh and dry weights), active compounds (ginsenosides Rg1, Re, Rf, Rg2, Rb1, Ro, Rc, Rb2, Rb3, and Rd), and rhizosphere soil fungal community. The main objectives of the study were the following: (i) to reveal changes in ginseng growth indexes and active compounds under nine fertilizer combinations to formulate precise fertilization schemes; (ii) to determine the diversity and community structure characteristics of rhizosphere fungi under each scheme; and (iii) to explore the responses of secondary metabolites in ginseng rhizomes to changes in the structure of soil fungal communities. The results will provide a basis for promoting growth, increasing yield, improving quality, and developing efficient cultivation of ginseng under scientifically validated fertilization management.

## Materials and Methods

### Site Description and Experimental Design

The experimental site was in the Qixing Baicao Garden at the Changchun University of Chinese Medicine in Changchun, Jilin Province, China (43°49′48^′′^N, 125°24′53^′′^E; altitude 399m). The region has a continental monsoon climate with an annual average temperature of 4.6°C and annual precipitation of ~600–700mm. In this study, an L_9_(3^4^) orthogonal experimental design was used to set three factors, namely, N, P, and K, at four levels for a total of 10 fertilization schemes, including an unamended control. Specific fertilization amounts are shown in Table 1. The experiment was conducted in pots (height: 11 cm; upper diameter: 12 cm; lower diameter: 9 cm; bottom with multiple drainage holes), with soil depth maintained at ~9 cm. Whole pots were buried in the soil so that the soil in the pots was in close contact with the ground, and the soil temperature was maintained to reduce the influence of temperature change on ginsenoside synthesis (Cheng et al., 2018; Wang et al., 2019). The test soil was a dark brown soil with a sandy loam texture and the following properties: pH, 6.38; organic matter, 49.02 g·kg^−1^; available N, 42.08 mg·kg^−1^; available P, 25.82 mg·kg^−1^; and available K, 107.50 mg·kg^−1^. On 17 May 2021, 2-year-old ginseng plants with good growth and relatively uniform size were selected and transplanted into the pots, with one plant per pot and each treatment with 20 replicate pots, for a total of 200 pots. After time for plant adaptation, 60 pots of 6 pots in each group were selected for one-time fertilization on 17 June 2021. Other management (such as protection measures, irrigation, and weeding) during cultivation was consistent with field measures. Fertilizer formulations were the following: urea (N, total N ≥ 46%), calcium superphosphate (P_2_O_5_, total P ≥ 46%), and potassium sulfate (K_2_O, total K ≥ 50%). For fertilization, the nine fertilizer combinations were dissolved in the same volume of water and evenly sprayed on the soil surface of pots.

**Table 1:** Types and amounts of fertilization in an L_9_(3^4^) orthogonal experimental design.

**Treatments**	**Groups**	**Application amount (g·m^−2^)**		
		**Urea (N)**	**Calcium superphosphate (P_**2**_O_**5**_)**	**Potassium sulfate (K_**2**_O)**
CK	N_0_P_0_K_0_	0	0	0
T1	N_1_P_1_K_1_	10	15	20
T2	N_1_P_2_K_2_	10	45	40
T3	N_1_P_3_K_3_	10	75	60
T4	N_2_P_1_K_2_	30	15	40
T5	N_2_P_2_K_3_	30	45	60
T6	N_2_P_3_K_1_	30	75	20
T7	N_3_P_1_K_3_	50	15	60
T8	N_3_P_2_K_1_	50	45	20
T9	N_3_P_3_K_2_	50	75	40

### Sample Collection and Determination of Growth Indexes

Ginseng rhizosphere soil was collected 90 days after fertilization. Rhizosphere soil was obtained after root-shaking. After removing surface soil, rhizosphere soil was collected by sterile brush and gentle shaking and then immediately stored at −80°C until analysis of fungi. Plant growth was determined by measuring three parameters: plant height, stem and leaf fresh weight, and rhizome fresh weight. Then, non-medicinal parts were removed, and rhizomes were cleaned, dried, weighed, and ground into a fine powder that passed through a 65-mesh (250μm) sieve. The powder was analyzed for ginsenoside contents.

### Determination of Active Compounds

The contents of ginsenosides were determined according to the method described by Bai et al. (2021), with slight modification. Medicinal material sample solutions were prepared according to the China Pharmacopoeia for HPLC-UV analysis (China Pharmacopoeia Committee, 2020). The contents of ginsenosides Rg1, Re, Rf, Rg2, Rb1, Ro, Rc, Rb2, Rb3, and Rd were determined on an Agilent EC-C18 column (4.6mm × 150mm; 2.7μm particle size). The column temperature was 40°C, the flow rate was 1 ml/min, and the mobile phase was acetonitrile (A) and 0.1% phosphoric acid aqueous solution (B). Gradient elution conditions were the following: 0–23min, 18% → 21% A; 23–35min, 21% → 28% A; 35–80min, 28% → 32% A. The UV detection wavelength was 203 nm, and the injection volume was 10 μl.

### DNA Extraction, PCR Amplification, and Illumina Sequencing

Rhizosphere soil samples were precisely weighed (0.5 g) and total DNA was extracted using an MN NucleoSpin Soil DNA kit (Macherey-Nagel, Düren, Germany). The concentration and purity of DNA were detected using a Multiskan Sky full-wavelength microplate reader (Thermo Fisher Scientific, Waltham, MA, USA), and then, DNA was stored at −20°C until further use. Fungal internal transcribed spacer (ITS) genes were amplified by PCR using the specific primers ITS1F (5′-CTTGGTTTAGAGGAAGTAA-3′) and ITS2 (5′-GCTGCGTTCTTCATCGATGC-3′) with bar codes. The reaction system included the following: template DNA, 50 ng; 10 μM forward primer, 0.3 μl; 10μM reverse primer, 0.3 μl; KOD FX Neo Buffer (TOYOBO, Osaka, Japan), 5 μl; KOD FX Neo, 0.2 μl; and 2mM dNTP, 2 μl; with ddH2O added to 10 μl. The reaction procedure was the following: 95°C pre-denaturation for 5min, 25 cycles (95°C for 30 s, 50°C for 30 s, and 72°C for 40 s), and final extension at 72°C for 5min. PCR products were quantified by electrophoresis (ImageJ Version 1.51) and mixed according to the mass ratio of 1:1. After mixing, an OMEGA DNA purification column (Omega Bio-Tek, Norcross, Georgia, USA) was used for column purification. Subsequently, paired-end sequencing (2 × 250) was performed on an Illumina HiSeq platform with an Illumina NovaSeq 6000 according to standard protocols. Sequencing of the ITS gene was performed by BioMarker Technologies Co., Ltd. (Beijing, China). All sequence data were submitted to the Sequence Read Archive (accession number: PRJNA828555) and are freely available at the NCBI (https://www.ncbi.nlm.nih.gov/sra/PRJNA828555).

### Data Processing and Bioinformatics Approaches

Data were pre-processed according to the following steps, and the species classification information corresponding to each feature was obtained. (i) Raw reads were filtered using Trimmomatic (Version 0.33). Then, Cutadapt (Version 1.9.1) was used to identify and remove primer sequences and obtain clean reads. (ii) Usearch (Version 10.0) was used to splice clean reads in each sample by overlap, and then the length of the spliced data was filtered according to the length range of different regions. (iii) UCHIME (Version 4.2) was used to identify and remove chimera sequences and obtain the final effective reads. (iv) Reads were clustered at 97.0% similarity using Usearch (Version 10.0) to obtain operational taxonomic units (OTUs) (Edgar, 2013). (v) With UNITE as the reference database (Release 7.2, http://unite.ut.ee/index.php), the naive Bayesian classifier was used to classify the feature sequences.

### Statistical Analyses

SPSS 21.0 and GraphPad Prism 8.0 were used for statistical analysis and graphic preparation, respectively, of growth indexes and active compound contents. A Student–Newman–Kells (SNK) post-special trial was used for multiple comparisons between different treatments, with a significance of *p* < 0.05. The principal component analysis (PCA) of Origin 2019 was used to evaluate the 10 fertilization schemes. Community richness and diversity indexes were calculated using QIIME (Version 1.9.1). The dilution curve was drawn by R software (Version 3.6.0), and differences in community composition were tested by Bray–Curtis distance. Principal coordinate analysis (PCoA) was conducted by WGCNA, stat, and ggplot2 packages in R software (Version 3.6.0). The vegan software package in R was used to generate heat maps to analyze the composition of fungal communities. Significant *p*-values in the LEfSe analysis were calculated using the Wilcoxon rank-sum test, and the LDA was set to 4.0. GraphPad Prism 8.0, Origin 2019, and R software (Version 3.6.0) were used to prepare images.

## Results

### Effects of NPK Combined Application on Growth Indexes of Ginseng

Growth characteristics reflect the adaptability of plants to the soil environment and are important indexes to evaluate plant vitality (Sun et al., 2022). Compared with CK, plant height increased in the fertilization treatments, except in T9, and the increases of 1.24-fold in T3 and 1.22-fold in T7 were significant (*p* < 0.05). The results were similar for stem and leaf fresh weight. The greatest stem and leaf biomass were in T3, but there were no significant differences among T3, T4, and T7 treatments (*p* > 0.05). Notably, rhizome fresh and dry weights were not the highest in T3. Instead, the greatest rhizome biomass was in T7, which did not have the highest aboveground biomass, and compared with CK, rhizome biomass increased by 43.65% (fresh weight) and 37.93% (dry weight).

### Effects of NPK Combined Application on Contents of Active Compounds in Ginseng

**Figure 1:**
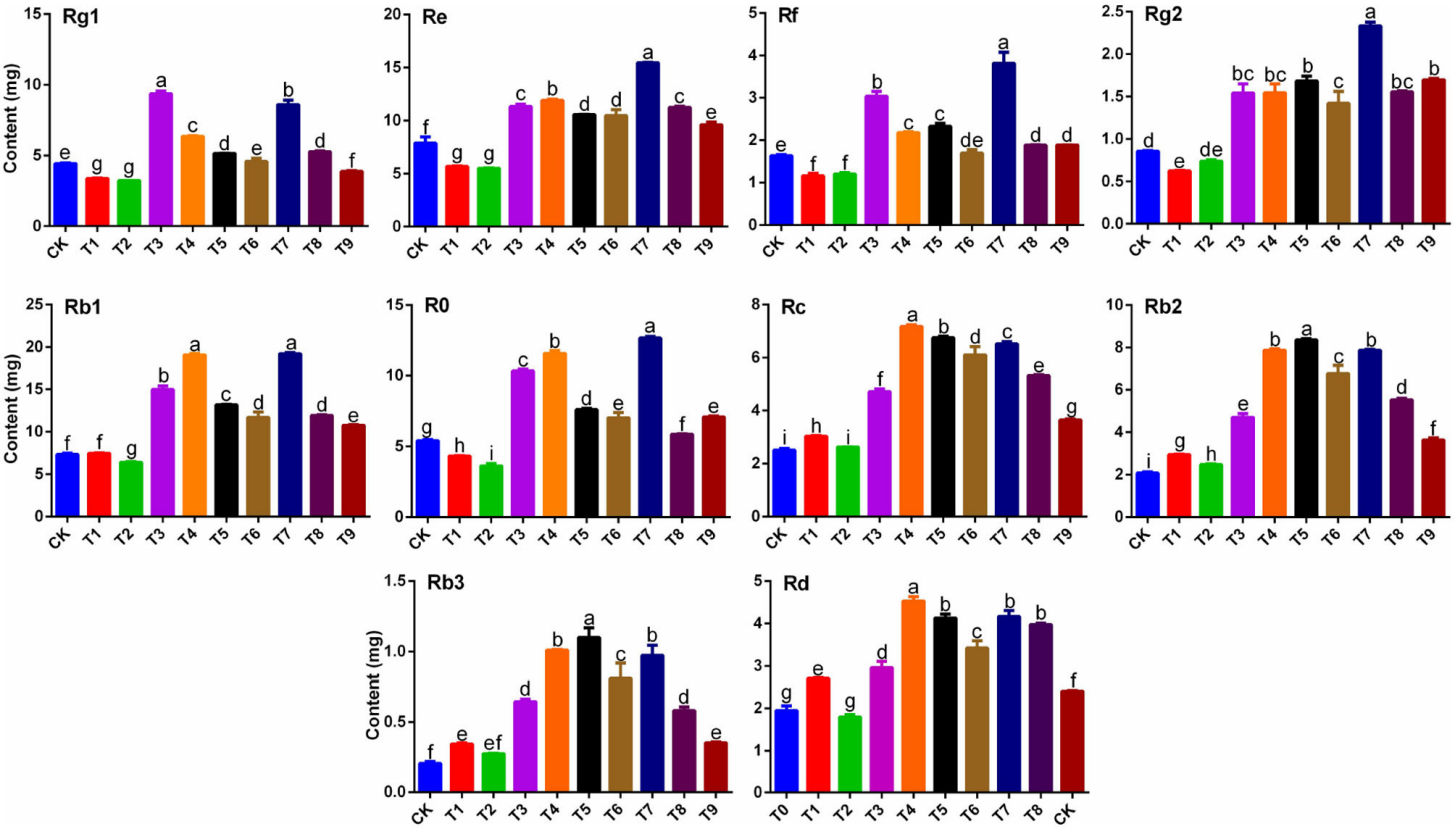
Effects of NPK combined application on contents of ginsenosides Rg1, Re, Rf, Rg2, Rb1, Ro, Rc, Rb2, Rb3, and Rd in ginseng rhizomes (*n* = 3). Note: (a) Different letters indicate significant differences among treatments at *p* < 0.05 according to SNK tests. (b) Composition of fertilization treatments: CK, N_0_P_0_K_0_; T1, N_1_P_1_K_1_; T2, N_1_P_2_K_2_; T3, N_1_P_3_K_3_; T4, N_2_P_1_K_2_; T5, N_2_P_2_K_3_; T6, N_2_P_3_K_1_; T7, N_3_P_1_K_3_; T8, N_3_P_2_K_1_; T9, N_3_P_3_K_2_.

Contents of 10 primary ginsenosides were determined (Figure 1) by analyzing the concentration of every single ginsenoside (Supplementary Figure S1) and the average rhizome weight (Table 2) under 10 fertilization schemes. Of the nine fertilizer combinations, T3 and T7 significantly increased the Rg1 content in ginseng rhizomes (*p* < 0.05) by 2.11-fold and 1.94-fold, respectively, compared with that in CK. Results for contents of ginsenoside Rf were similar to those of Rg1, and Rf contents in T3 and T7 were significantly higher than those in other treatments (*p* < 0.05). In contrast to Rg1, Rf content was highest in T7, with the content 2.33-fold higher than that in CK. Contents of ginsenosides Re and Rg2 were also significantly higher in T7 than in other treatments (*p* < 0.05), with contents 95.87 and 172.77% higher, respectively, than those in CK. Contents of ginsenosides Rb1 and Ro showed similar patterns among the nine fertilizer combinations, with the highest contents in T4 and T7. Notably, there were striking similarities in the patterns of contents of Rc, Rb2, Rb3, and Rd in the nine fertilizer combinations. Except in T2, contents of the four ginsenosides were significantly higher in other treatments than in CK (*p* < 0.05), with the highest contents in T4, T5, and T7.

**Table 2:** Effects of NPK combined application on growth indexes of ginseng.

**Treatments**	**Plant height (cm)**	**Stem and leaf fresh weight (g)**	**Rhizome fresh weight (g)**	**Rhizome dry weight (g)**
CK	57.57 ± 2.23b	4.40 ± 0.46c	7.17 ± 0.99c	2.03 ± 0.18bc
T1	58.93 ± 6.50b	5.13 ± 0.55bc	7.74 ± 1.00bc	1.51 ± 0.18cd
T2	58.67 ± 1.60b	5.64 ± 0.75b	7.93 ± 0.46bc	1.35 ± 0.52d
T3	71.33 ± 4.51a	7.47 ± 0.49a	9.70 ± 0.74ab	2.61 ± 0.13ab
T4	64.13 ± 3.68ab	6.95 ± 0.43a	10.17 ± 0.82a	2.38 ± 0.10ab
T5	60.83 ± 3.62b	5.70 ± 0.45b	8.28 ± 0.86abc	2.02 ± 0.19bc
T6	58.67 ± 5.86b	5.30 ± 0.41bc	7.21 ± 0.55c	1.96 ± 0.40bc
T7	70.33 ± 2.52a	7.09 ± 0.28a	10.30 ± 0.67a	2.80 ± 0.26a
T8	64.17 ± 2.57ab	6.05 ± 0.52b	8.79 ± 1.20abc	2.29 ± 0.22ab
T9	54.33 ± 3.79b	4.93 ± 0.41bc	7.41 ± 1.28c	2.21 ± 0.13ab

*Note: (a) Different letters in a column indicate significant differences at p < 0.05, according to SNK tests (n = 6). (b) Initial growth indexes of ginseng seedlings: no aboveground indexes; rhizome fresh weight, 2.75 ± 0.80 g. (c) Composition of fertilization treatments: CK, N_0_P_0_K_0_; T1, N_1_P_1_K_1_; T2, N_1_P_2_K_2_; T3, N_1_P_3_K_3_; T4, N_2_P_1_K_2_; T5, N_2_P_2_K_3_; T6, N_2_P_3_K_1_; T7, N_3_P_1_K_3_; T8, N_3_P_2_K_1_; T9, N_3_P_3_K_2_*.

### Effects of NPK Combined Application on the Accumulation of Active Compounds in Ginseng

**Figure 2:**
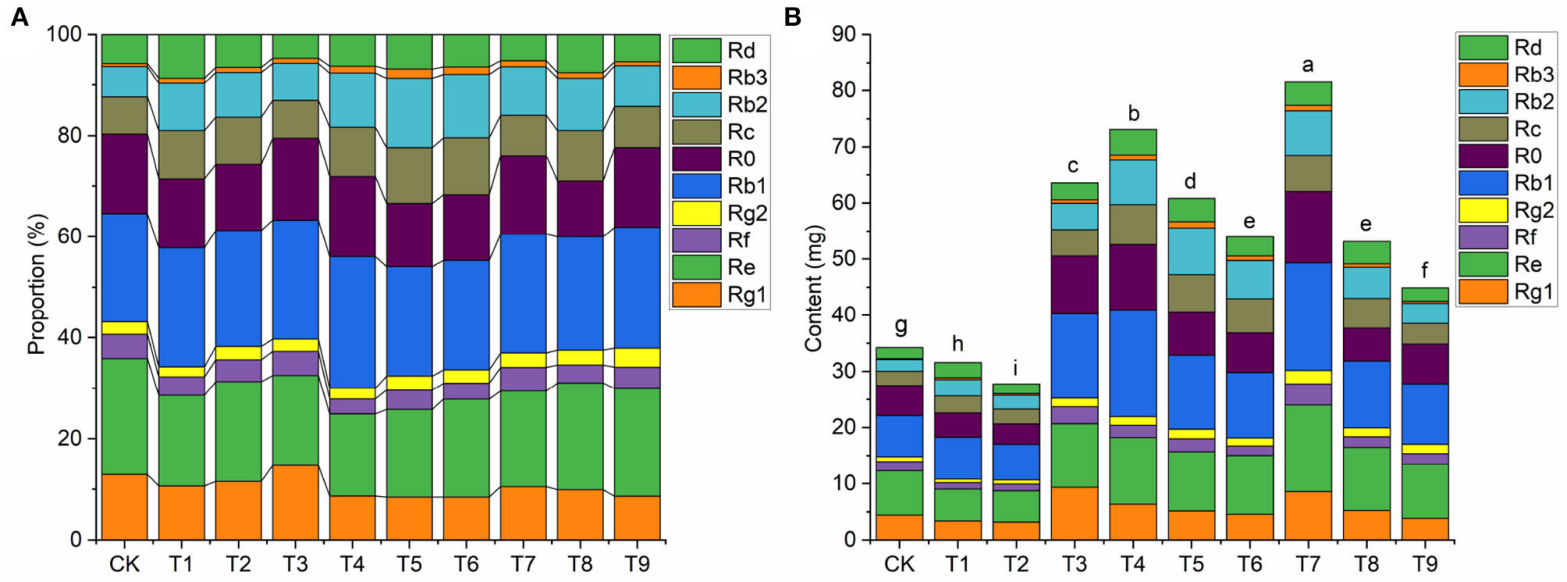
Effects of NPK combined application on **(A)** proportions (%) of ginsenosides and **(B)** total content (mg) of ginsenosides in ginseng (*n* = 3). Note: (a) Different letters indicate significant differences in total amounts at *p* < 0.05 according to SNK tests. (b) Composition of fertilization treatments: CK, N_0_P_0_K_0_; T1, N_1_P_1_K_1_; T2, N_1_P_2_K_2_; T3, N_1_P_3_K_3_; T4, N_2_P_1_K_2_; T5, N_2_P_2_K_3_; T6, N_2_P_3_K_1_; T7, N_3_P_1_K_3_; T8, N_3_P_2_K_1_; T9, N_3_P_3_K_2_.

The proportion and contents of active ingredients are important indicators of daodi medicinal materials (Peng et al., 2021). Under different combinations of NPK, proportions of the 10 ginsenosides changed significantly (Figure 2A, Supplementary Table S1). As an example, compared with CK, proportions of Rb1 and Rb2 increased notably in T7, whereas those of Rg1 and Re decreased slightly. The accumulation of active ingredients directly determines the quality of medicinal materials. Contents of total ginsenosides (Figure 2B) in the nine treatments followed a pattern similar to that of rhizome biomass (Table 2). The total ginsenoside content was significantly higher in T7 than in the other treatments (*p* < 0.05), and the total ginsenoside content per plant reached 81.55mg, which was 2.38-fold higher than that in CK.

### Analysis of Optimum Fertilization Scheme for Ginseng

Principal component analysis was used to comprehensively evaluate the biomass and ginsenoside contents of ginseng rhizomes in 10 fertilization treatments at harvest. The PC1 and PC2 scores accounted for 91.8% of the total variance, with PC1 accounting for 80.5% and PC2 accounting for 11.3%. Therefore, differences among treatments were primarily controlled by PC1. Similar to total ginsenoside accumulation, the highest comprehensive score was in T7 (N_3_P_1_K_3_), with that of T4 (N_2_P_1_K_2_) slightly lower. Compared with CK, treatments with NPK applications tended to shift to the right along PC1. The T4 treatment [(N_2_P_1_K_2_): N fertilizer, 30 g·m^−2^; P fertilizer, 15 g·m^−2^; K fertilizer, 40 g·m^−2^] promoted the accumulation of the monomer ginsenosides Rc, Rb2, Rb3, and Rd. The T7 treatment [(N_3_P_1_K_3_): N fertilizer, 50 g·m^−2^; P fertilizer, 15 g·m^−2^; K fertilizer, 60 g·m^−2^] effectively promoted the accumulation of ginseng rhizome biomass, total ginsenosides, and the monomer ginsenosides Rg1, Re, Rf, Rg2, Rb1, and Ro.

### NPK Combined Application Affected Fungal Community Diversity

An Illumina Novaseq platform was used for high-throughput sequencing of 30 soil samples. A total of 2,390,934 clean reads were obtained after quality control filtration, with an average of 79,698 reads per sample. A total of 1,846 fungal OTUs were identified with clustering at 97.0% similarity. The sparse curve showed that the number of OTUs was close to saturation, indicating that sequencing was sufficient to reflect the real conditions of samples (Supplementary Figure S2).

The specific sequencing results are shown in Table 3. The range of clean reads of samples was from 79,478 to 79,977, and the range of OTUs was from 1,499 to 1,750. Good's coverage of all samples was >99.80%. Alpha diversity reflects species richness and diversity of samples. The Shannon and Simpson indexes were used to measure species diversity, and the Chao1 and ACE indexes were used to estimate species richness. Compared with CK, the Shannon diversity index of ginseng rhizosphere fungal communities increased in the nine fertilizer treatments. The highest Shannon diversity index was in T9, which was significantly higher than that in CK (*p* < 0.05). The results indicated that the application of NPK increased the diversity of fungal communities. In contrast to the Shannon index, the Simpson index was not significantly different among the 10 fertilization schemes (*p*>0.05). The Chao1 and ACE indexes had similar trends in the 10 fertilization treatments. Indexes in T1, T2, and T9 were significantly higher than those in CK, whereas indexes in T3–T8 were significantly lower than those in CK (*p* < 0.05). The results indicate that a precise fertilization scheme is a key to regulate the structure of fungal communities and create a suitable soil microenvironment.

**Table 3:** High-throughput sequencing results and α-diversity indexes of fungal communities in ginseng rhizosphere soil samples.

**Treatments**	**Clean reads**	**OTUs**	**Good's_ coverage**	**Shannon**	**Simpson**	**Chao1**	**ACE**
CK	79478 ± 398	1556 ± 79	0.998	8.06 ± 0.24c	0.9881 ± 0.0039a	1657.17 ± 63.42c	1634.02 ± 70.05b
T1	79745 ± 62	1662 ± 35	0.998	8.39 ± 0.06ab	0.9913 ± 0.0006a	1742.47 ± 37.58b	1722.19 ± 38.87a
T2	79547 ± 184	1663 ± 13	0.998	8.3 ± 0.21bc	0.9889 ± 0.0037a	1717.91 ± 11.21b	1708.32 ± 16.03a
T3	79728 ± 82	1507 ± 15	0.999	8.32 ± 0.03bc	0.9872 ± 0.0006a	1548.76 ± 15.36d	1530.03 ± 16.58c
T4	79604 ± 149	1509 ± 20	0.999	8.34 ± 0.04ab	0.9872 ± 0.0006a	1546.62 ± 27.79d	1530.64 ± 21.84c
T5	79838 ± 132	1499 ± 20	0.999	8.2 ± 0.04bc	0.9872 ± 0.0004a	1554.47 ± 26.2d	1532.58 ± 20.54c
T6	79977 ± 255	1512 ± 19	0.999	8.43 ± 0.03ab	0.9889 ± 0.0003a	1539.69 ± 24.19d	1530.81 ± 22.75c
T7	79577 ± 33	1502 ± 8	0.999	8.28 ± 0.02bc	0.9867 ± 0.0005a	1539.47 ± 4.17d	1524.59 ± 7.47c
T8	79760 ± 90	1526 ± 37	0.999	8.47 ± 0.05ab	0.9893 ± 0.0003a	1569.89 ± 42d	1549.64 ± 38.1c
T9	79723 ± 78	1750 ± 46	0.999	8.6 ± 0.03a	0.9915 ± 0.0007a	1801.87 ± 17.29a	1775.52 ± 32.46a

*Note: (a) Different letters in a column indicate significant differences at p < 0.05 according to SNK tests (n = 3). (b) Composition of fertilization treatments: CK, N_0_P_0_K_0_; T1, N_1_P_1_K_1_; T2, N_1_P_2_K_2_; T3, N_1_P_3_K_3_; T4, N_2_P_1_K_2_; T5, N_2_P_2_K_3_; T6, N_2_P_3_K_1_; T7, N_3_P_1_K_3_; T8, N_3_P_2_K_1_; T9, N_3_P_3_K_2_*.

### NPK Combined Application Affected Fungal Community Composition

Fungal communities were classified and identified based on OTUs. The proportions of fungal taxa in ginseng rhizosphere soil were notably different in the nine fertilizer treatments. Ascomycota, Mortierellomycota, Basidiomycota, Glomeromycota, and Chytridiomycota were the main phyla of fungi in ginseng rhizosphere soil (**Figure 4A**). Relative abundances of the 10 most abundant fungal phyla in each treatment are shown in Supplementary Table S2. The relative abundance of Ascomycota was highest in CK (79.25%). With fertilization, relative abundances of Ascomycota decreased compared with that in CK. In contrast to Ascomycota, relative abundances of Mortierellomycota, Basidiomycota, Glomeromycota, and Chytridiomycota increased after fertilization compared with those in CK. Relative abundances of Mortierellomycota and Glomeromycota in each fertilization treatment were notably different from those in CK, with the highest abundances in T7 of 16.33 and 4.06%, respectively.

The 10 most abundant genera of fungi in ginseng rhizosphere soil were *Mortierella, Fusarium, Aspergillus, Chaetomium, Purpureocillium, Cladosporium, Penicillium, Monascus, Botryotrichum*, and *Metacordyceps* (**Figure 4B**). The relative abundances of dominant fungal genera are listed in Supplementary Table S3. The nine fertilizer combinations changed the relative abundances of genera. Compared with CK, the relative abundances of *Mortierella, Chaetomium, Purpureocillium*, and *Metacordyceps* in the fertilization treatments increased. The increases in the relative abundance of those genera indicated that fertilization could increase the richness of potential beneficial fungi in ginseng rhizosphere soil. The potential beneficial genera of fungi increased notably in T3, T4, and T7, with total relative abundances of 26.20, 26.12, and 26.14%, respectively. Notably, ginseng rhizosphere soils contained high relative abundances of *Fusarium*, which is a common pathogen that can infect plants and cause many diseases, such as plant root rot (Li et al., 2018). Compared with CK, NPK combined applications effectively decreased relative abundances of *Fusarium* and thus increased the suitability of the soil microenvironment for ginseng growth and development. Similarly, relative abundances of *Aspergillus, Cladosporium, Penicillium, Monascus*, and *Botryotrichum* decreased after fertilization compared with those in CK. Cumulative decreases in potential harmful fungi were relatively greater in T3, T4, and T7 than in other treatments, and in those treatments, relative abundances were 12.32, 12.30, and 12.05% lower, respectively, than those in CK.

### NPK Combined Application Affected Fungal Community Structure

To compare the fungal community structure in the 10 fertilization treatments, PCA and PCoA analyses were performed. The two-dimensional PCA diagram based on OTUs showed clear differences among different treatments (**Figure 5A**). The extracted two main coordinates explained 82.21% of the variation, with PC1 explaining 69.71% of the variation and PC2 explaining 12.50% of the variation. Compared with CK, the fertilization treatments were farther to the left on PC1, with T7, T4, and T3 farthest to the left, followed by T5, T6, and T8. Samples in treatments T1, T2, and T9 were not separated from CK.

The PCoA analysis based on independent OTU unweighted binary_jaccard distance also showed notable differences in fungal community structure among different treatments (**Figure 5B**). The first two main coordinates of the PCoA explained 38.49% (PC1) and 4.98% (PC2) of the total variation in the fungal community structure. Similar to the PCA, CK, T1, T2, and T9 formed a group on the side of the coordinate axis, whereas treatments T3–T8 formed another group. The two analyses showed that the fungal community structure of ginseng rhizosphere soil was affected by differences in the nine fertilizer combinations.

To further explore differences in the fungal community structure among the 10 fertilization schemes, a heat map of the relative abundances of the 50most abundant genera was prepared (**Figure 6**). There were clear changes in fungal community structure among treatments according to relative proportion values and color changes in the heat map. Fungal community composition was similar in two different groups of treatments: T1, T2, T9, and CK, and treatments T3–T8, which was a result consistent with those of the PCA and PCoA.

### Analysis of the Dominant Fungal Community Under the Optimum Fertilization Scheme

To further identify structural differences in fungal communities between CK and the optimal fertilization scheme T7, LEfSe analysis was conducted on the two treatments, and the community differences from phylum to genus were explored (**Figure 7**). At the phylum level, Ascomycota was abundant in CK, whereas Mortierellomycota and Glomeromycota were abundant in T7. At class, order, and family levels, in T7, the abundant rhizosphere taxa were Mortierellomycetes (class), Agaricomycetes (class), Glomeromycetes (class), Mortierellales (order), Glomerales (order), Mortierellaceae (family), Glomeraceae (family), and Ophiocordycipitaceae (family). Seven genera were identified in CK and T7, among which three were considered biomarkers of the T7 treatment, namely, *Mortierella, Chaetomium*, and *Purpureocillium*. In CK, four genera were identified as biomarkers, which included *Aspergillus, Botryotrichum, Fusarium*, and *Penicillium*.

### Correlation Analysis of Key Rhizosphere Fungal Groups With Ginseng Yield and Ginsenoside Contents

To examine relations between ginseng yield and quality and fungal communities, Spearman correlation analyses were performed on dry and fresh weights of rhizomes; contents of ginsenosides Rg1, Re, Rf, Rg2, Rb1, Ro, Rc, Rb2, Rb3, and Rd; and relative abundances of fungal genera *Mortierella, Fusarium, Aspergillus, Chaetomium, Purpureocillium, Cladosporium, Penicillium, Monascus, Botryotrichum*, and *Metacordyceps* (**Figure 8**).

There were significant positive correlations between the contents of the 10 primary ginsenosides in ginseng rhizomes. There were also significant positive correlations between total ginsenoside content and fresh and dry weights of rhizomes, with correlation coefficients of 0.72 and 0.81, respectively. The 10 main fungal genera in ginseng rhizosphere soil were notably different. Of those genera, *Mortierella, Chaetomium, Purpureocillium*, and *Metacordyceps* were positively correlated, with correlation coefficients that were >0.70. Notably, those four genera were significantly negatively correlated with the other six genera of fungi, indicating strong antagonism between the two fungal groups.

Relative abundances of *Mortierella, Chaetomium, Purpureocillium*, and *Metacordyceps* were positively correlated with the accumulation of individual ginsenosides. The four genera were also significantly positively correlated with the total ginsenoside content, with correlation coefficients of 0.92, 0.84, 0.67, and 0.89, respectively. They were also positively correlated with fresh and dry weights of rhizomes, indicating the four genera were associated with the growth of ginseng and the synthesis of active compounds. By contrast, relative abundances of *Fusarium, Aspergillus, Cladosporium, Penicillium, Monascus*, and *Botryotrichum* were negatively correlated with ginsenoside contents and rhizome biomass, indicating they were associated with decreases in accumulation of ginseng rhizome biomass and ginsenoside biosynthesis.

## Discussion

### Insights Into Effects of NPK Fertilization on Ginseng Growth and Quality

Scientifically precise NPK fertilization as an advanced technology to improve soil quality and improve agronomic productivity has gradually been seriously considered (Wang et al., 2020). The technology not only improves crop yield but also the quality, which greatly promotes research on nutrients in plant–soil systems (Awan et al., 2022). In this study, precise NPK application rates and proportions were key in increasing ginseng growth and development. First, the growth of ginseng aboveground parts (i.e., plant height and stem and leaf fresh weight) showed high adaptability and responsiveness to the N_1_P_3_K_3_ (T3) treatment, which is consistent with applications of P and K fertilizers as keys to the vigorous growth of leaves on plant stems (Sun et al., 2022). Second, ginseng belowground biomass (i.e., rhizome fresh and dry weights) was also analyzed because rhizome biomass directly determines yields of medicinal materials. Rhizome fresh and dry weights were greater in the N_3_P_1_K_3_ (T7) fertilization scheme than in the other fertilization treatments. Therefore, the combined effects of moderate amounts of N and K in low P soil notably increased the development of ginseng roots.

When planting medicinal materials, the focus should be on both yield and quality (Ostadi et al., 2020). In this study, sufficient amounts of NPK fertilizers increased the accumulation of secondary metabolites in ginseng. However, the level of response of each ginsenoside monomer to the nine fertilizer combinations was different. Five monomer ginsenosides (Re, Rf, Rg2, Rb1, and Ro) responded positively to the N_3_P_1_K_3_ (T7) treatment. In N_2_P_1_K_2_ (T4) and N_2_P_2_K_3_ (T5) treatments, the contents of two monomer ginsenosides increased. The monomer Rg1 specifically responded in the N_1_P_3_K_3_ (T3) scheme. In a comprehensive analysis of the 10 monomer ginsenosides (**Figure 2**), the accumulation of total ginsenosides increased significantly in the N_3_P_1_K_3_ (T7) treatment. Based on the results, formulating a targeted fertilization scheme can effectively promote the accumulation of one or more components in medicinal plants, which also supports the further development of precision agriculture.

**Figure 3:**
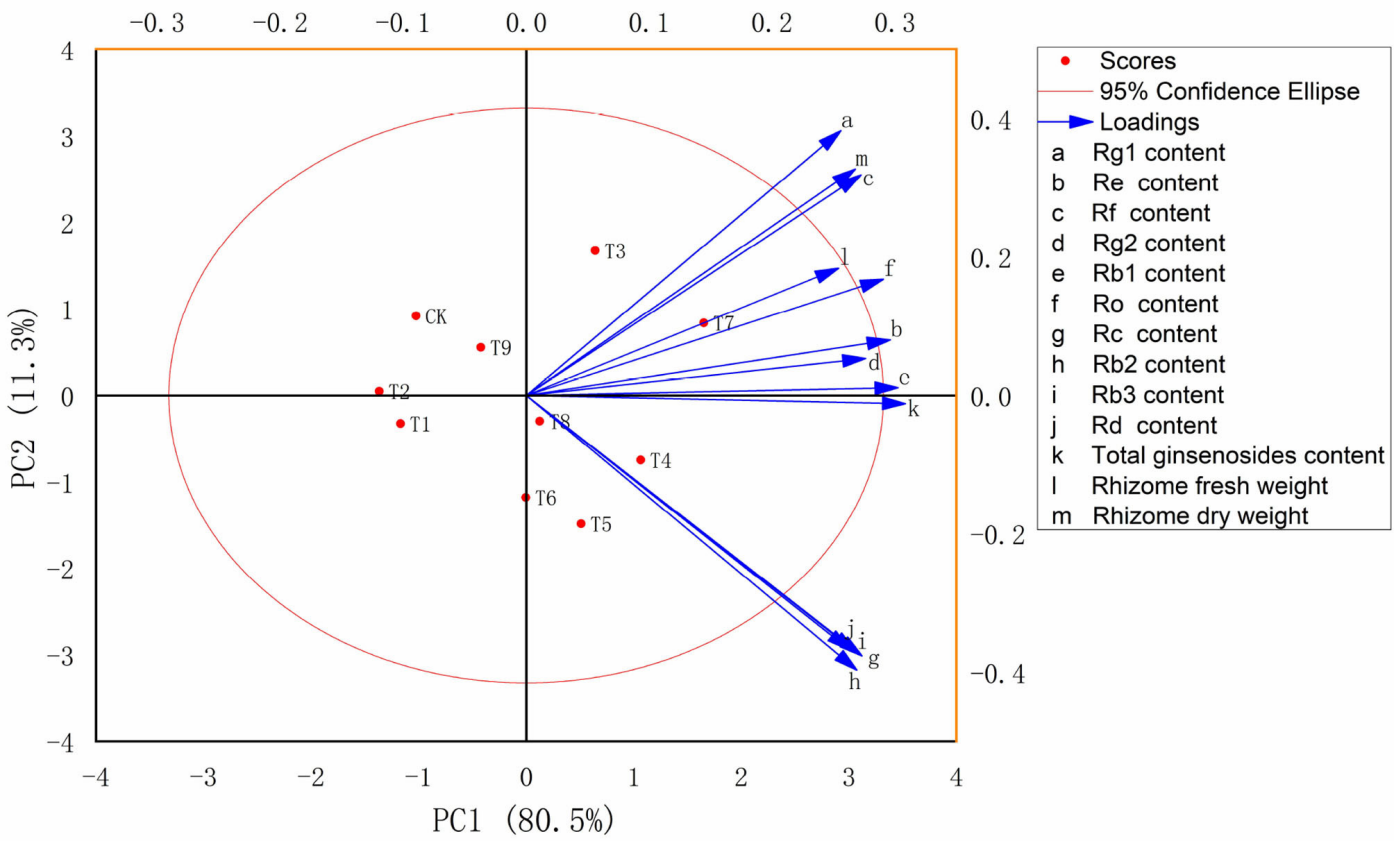
Multi-index principal component (PC) analysis score map of ginseng in 10 fertilization treatments (*n* = 10). Note: (a) Composition of fertilization treatments: CK, N_0_P_0_K_0_; T1, N_1_P_1_K_1_; T2, N_1_P_2_K_2_; T3, N_1_P_3_K_3_; T4, N_2_P_1_K_2_; T5, N_2_P_2_K_3_; T6, N_2_P_3_K_1_; T7, N_3_P_1_K_3_; T8, N_3_P_2_K_1_; T9, N_3_P_3_K_2_.

To further examine relations between physiological properties and soil nutrients in the soil–plant system, PCA was performed on ginseng yield and quality indexes in the 10 fertilization schemes (Figure 3). According to the PCA, the N_3_P_1_K_3_ (T7) treatment had the most comprehensive benefits. Therefore, N_3_P_1_K_3_ (T7) was the optimal fertilizer combination to improve ginseng yield and quality. Because the scheme of low P and high N and K amounts of fertilizer obtained the greatest benefits in ginseng cultivation, N fertilizer at 500 kg·ha^−1^, P fertilizer at 150 kg·ha^−1^, and K fertilizer at 600 kg·ha^−1^ are recommended in agricultural production. Optimizing NPK composition to achieve efficient and sustainable agricultural production appears to involve complex processes that are entirely dependent on the harmonious behavior of the plant–soil system (Wei et al., 2018; Sun et al., 2022). However, to balance agricultural development and ecological protection, such optimization is also necessary. In addition, this study provides new insights into optimizing ginseng fertilization regimens for precise addition of NPK in chemical fertilizers with defined ratios/compositions according to fertilization characteristics, which will be reliable and cost-effective.

### Insights Into Effects of NPK Fertilization on Rhizosphere Fungal Communities of Ginseng

High-throughput sequencing of fungal ITS gene markers has greatly increased understanding of soil nutrient–microorganism relationships by providing comprehensive descriptions of soil fungal community structure and diversity (Cai et al., 2021). In agricultural production, land use and management, such as quality and quantity of soil nutrient inputs, directly affect the composition of root-associated fungal communities (Pan et al., 2020). In this study, α diversity of ginseng rhizosphere fungi increased under NPK combined application, but the richness was bidirectionally regulated by NPK proportion and dose (Table 3).

**Figure 4:**
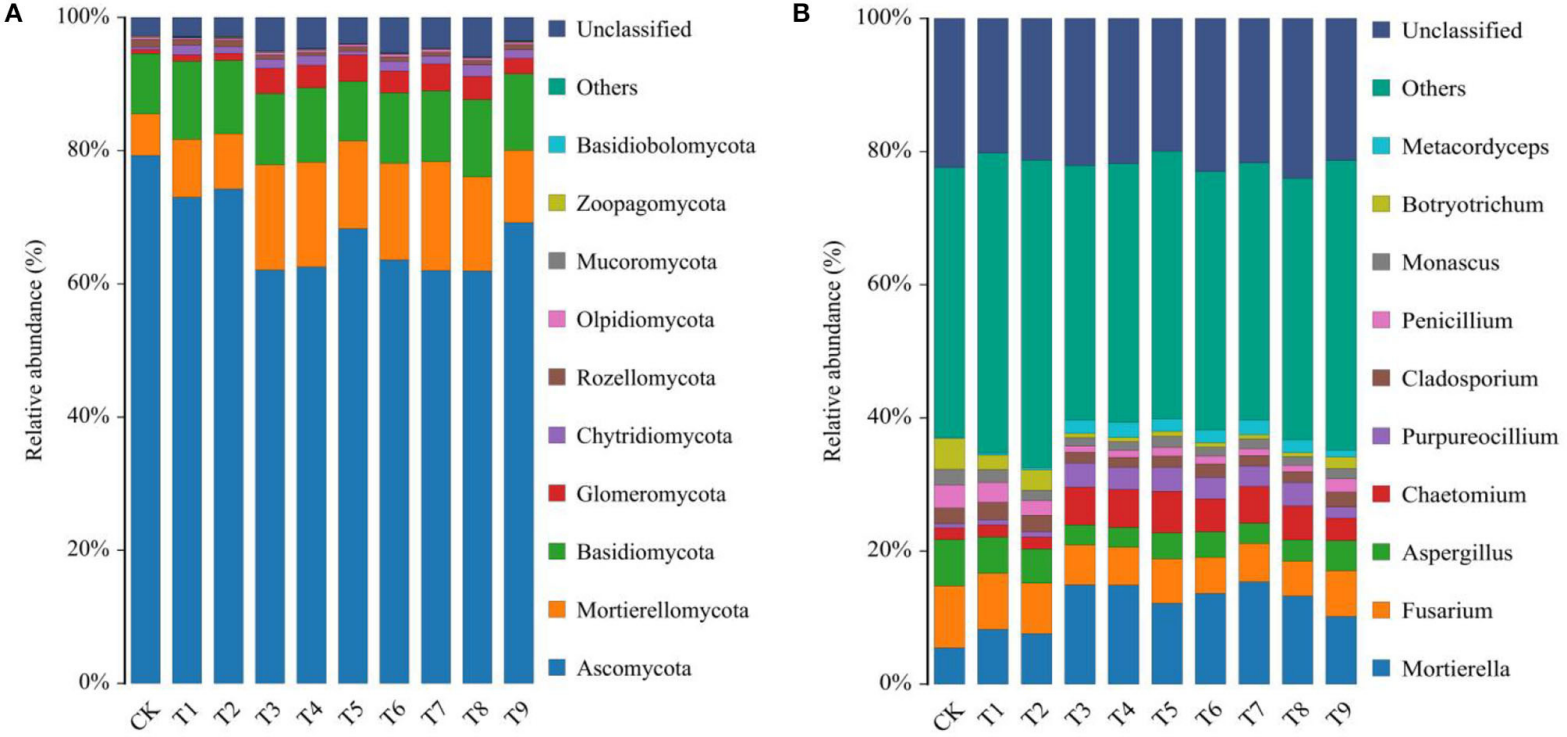
Relative abundance of fungal, **(A)** phyla, and **(B)** genera in rhizosphere soils of ginseng in 10 fertilization treatments. Note: (a) Composition of fertilization treatments: CK, N_0_P_0_K_0_; T1, N_1_P_1_K_1_; T2, N_1_P_2_K_2_; T3, N_1_P_3_K_3_; T4, N_2_P_1_K_2_; T5, N_2_P_2_K_3_; T6, N_2_P_3_K_1_; T7, N_3_P_1_K_3_; T8, N_3_P_2_K_1_; T9, N_3_P_3_K_2_.

The diversity and composition of fungal communities are important in maintaining ecosystem balance (Wang et al., 2021). Ascomycota was the dominant phylum of fungi in ginseng rhizosphere soil (Tong et al., 2021), and its relative abundance decreased under NPK combined application (Figure 4). The second most abundant phylum was Mortierellomycota, and its relative abundance increased notably after fertilization. Mortierellomycota was closely associated with soil nutrients, and the phylum was considered to be a marker group in the rhizosphere soil of high-quality ginseng (Fang et al., 2022). Glomeromycota also increased after fertilization, and its relative abundance was the highest in the N_3_P_1_K_3_ (T7) treatment. The phylum contains important root symbiotic fungi, which benefit plants by increasing soil nutrient uptake in exchange for photosynthetically fixed carbon (Malar et al., 2022). In this study, the application of NPK fertilizers reduced the relative abundance of Ascomycota in soil and increased the relative abundance of Mortierellomycota and Glomeromycota. Excitingly, other researchers have also confirmed the similar effects of chemical fertilizers on soil fungi (Zhang et al., 2021b; Su et al., 2022).

At the genus level, NPK combined application increased relative abundances of *Mortierella, Chaetomium, Purpureocillium*, and *Metacordyceps*, which were considered as possible potential beneficial fungi that could coexist in harmony with plants. *Mortierella* is widely found in the rhizosphere soils of agroforestry systems in the temperate zone. The genus can inhibit the growth of many potential pathogenic microorganisms by producing antibiotics and competing for nutrients and is also important in maintaining the microbial ecological balance (Tagawa et al., 2010). Meanwhile, *Mortierella* can stimulate the dissolution of insoluble phosphorus in soil by secreting oxalic acid (Osorio and Habte, 2013), which is presumed to be the main reason for its high relative abundance in a low P environment. *Chaetomium* can improve crop root quality, and because it antagonizes pathogens, the genus can be used for biological control (Ma et al., 2021). *Purpureocillium* and *Metacordyceps* are important in insect and nematode control (Elsherbiny et al., 2021). Notably, copiotrophic fungi, such as *Mortierella* and *Chaetomium*, as an important link between plants and soil, can affect the material cycle and energy conversion process in the soil environment through fierce competition for carbon sources (Zhao et al., 2019). The change in their relative abundance, as a more intuitive manifestation of changes in soil biochemical components, often also marks the emergence of some soil problems, such as the lack of other microorganisms that are important to the structure and function of soil ecosystems (Zhao et al., 2018). By contrast, relative abundances of the other six major genera of fungi, represented by *Fusarium*, decreased after fertilization. *Fusarium* is the main cause of ginseng root rot that causes poor growth and yield loss (Li et al., 2018). In a comprehensive comparison of the 10 fertilization schemes, treatments N_1_P_3_K_3_ (T3), N_2_P_1_K_2_ (T4), and N_3_P_1_K_3_ (T7) effectively increased relative abundances of potential beneficial fungi in rhizosphere soil while also notably decreasing those of potentially harmful fungi.

**Figure 5:**
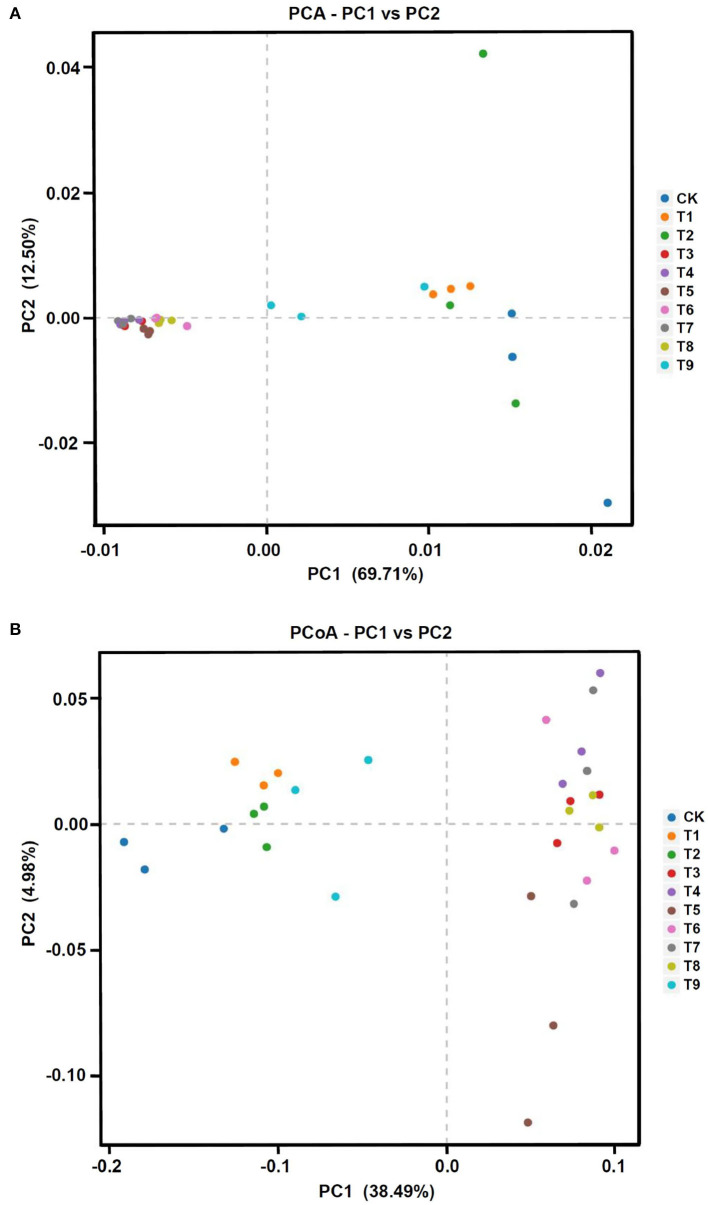
Fungal community structure in ginseng rhizosphere soil in different NPK fertilization treatments. **(A)** Principal component analysis (PCA) and **(B)** principal coordinate analysis (PCoA) of fungal communities based on operational taxonomic units. Note: (a) Composition of fertilization treatments: CK, N_0_P_0_K_0_; T1, N_1_P_1_K_1_; T2, N_1_P_2_K_2_; T3, N_1_P_3_K_3_; T4, N_2_P_1_K_2_; T5, N_2_P_2_K_3_; T6, N_2_P_3_K_1_; T7, N_3_P_1_K_3_; T8, N_3_P_2_K_1_; T9, N_3_P_3_K_2_.

**Figure 6:**
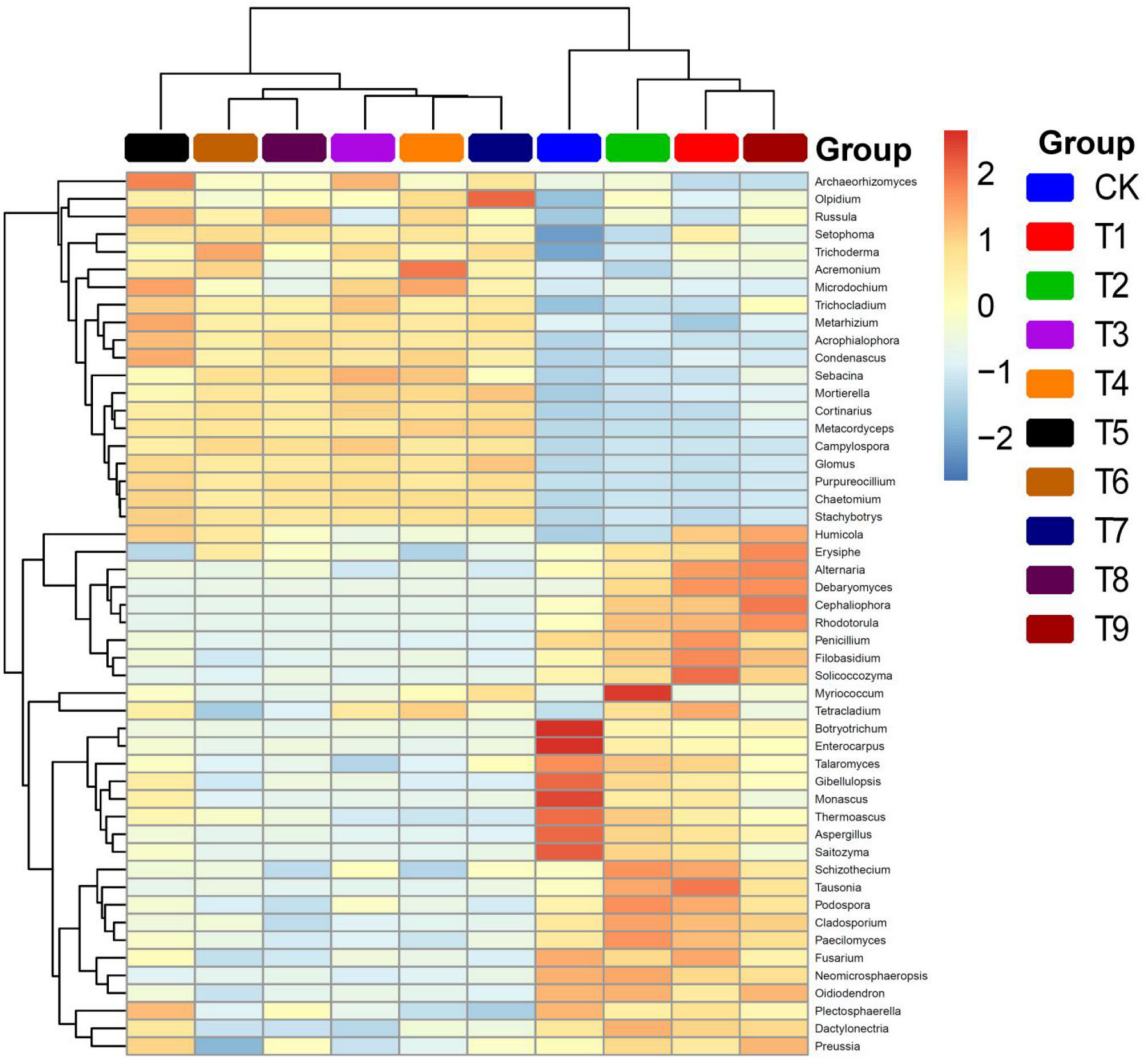
Heat map of relative abundances of genera in ginseng rhizosphere fungal communities in different NPK fertilizer treatments. Note: (a) Composition of fertilization treatments: CK, N_0_P_0_K_0_; T1, N_1_P_1_K_1_; T2, N_1_P_2_K_2_; T3, N_1_P_3_K_3_; T4, N_2_P_1_K_2_; T5, N_2_P_2_K_3_; T6, N_2_P_3_K_1_; T7, N_3_P_1_K_3_; T8, N_3_P_2_K_1_; T9, N_3_P_3_K_2_.

**Figure 7:**
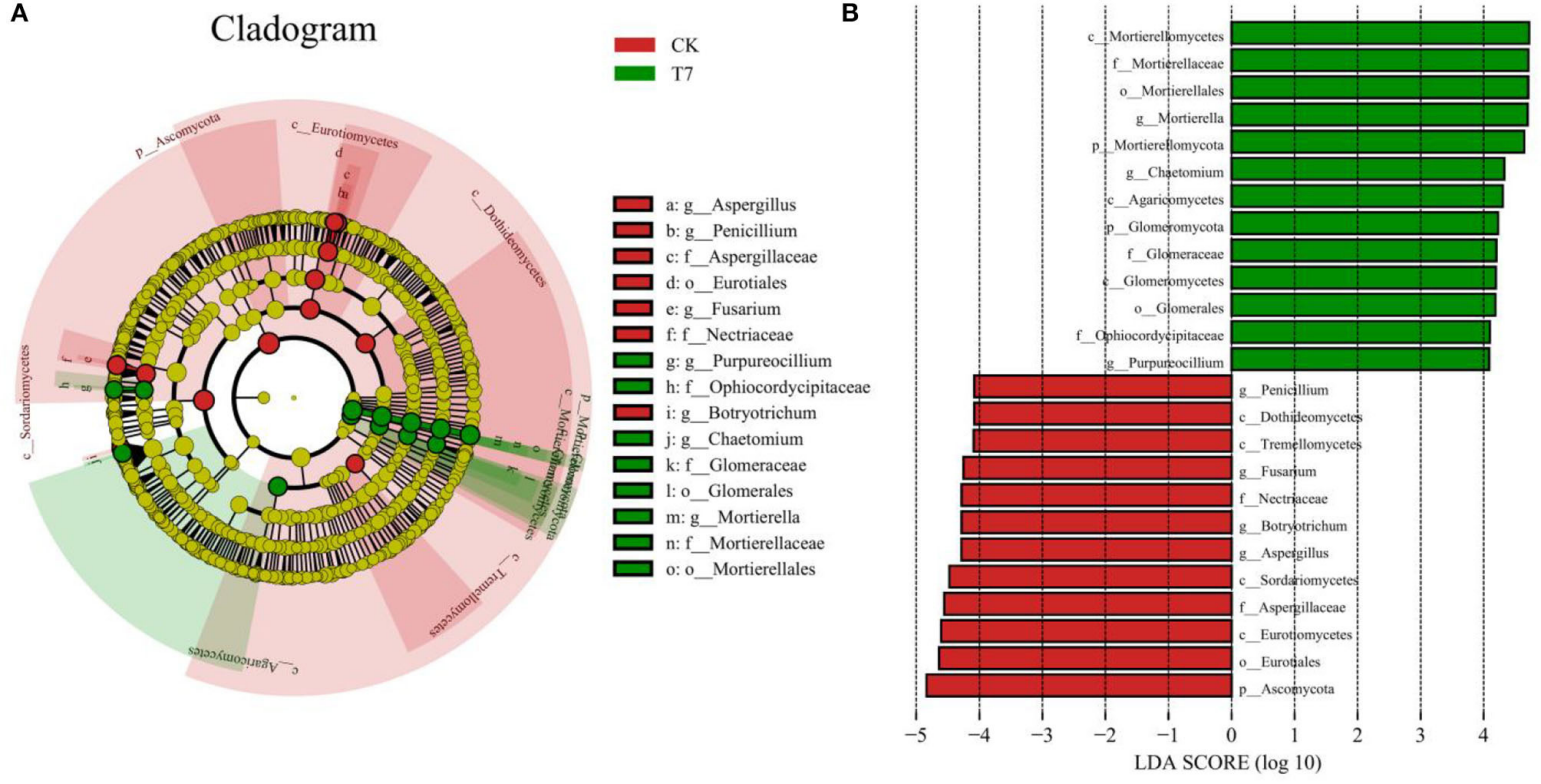
LEfSe comparison of fungal communities between rhizosphere soils of the control (CK) and the optimal fertilization treatment T7. **(A)** Cladogram of taxonomic levels from phylum to genus and **(B)** taxa with LDA values > 4. Note: (a) Composition of fertilization treatments: CK, N_0_P_0_K_0_; T7, N_3_P_1_K_3_.

In analyses of fungal community structure in ginseng rhizosphere soils (Figures 5, 6), treatments N_1_P_3_K_3_ (T3), N_2_P_1_K_2_ (T4), and N_3_P_1_K_3_ (T7) were clustered into one group. In the dimension reduction analysis, a large distance from the control group was always maintained, which was consistent with the changes in yield and quality. However, different fertilization schemes could shape similar fungal communities, and the yield and quality of ginseng under the synergistic effect of fertilizers and fungi was different, indicating that precise NPK application was the main factor for high yield and quality of crops. In combination with the previous comprehensive evaluation of each fertilization scheme (Figure 3), LEfSe analysis was used to further clarify the structural differences between CK and the optimal fertilization scheme N_3_P_1_K_3_ (T7) (Figure 7). The biomarkers in the two groups were an indication of the positive effect of the T7 scheme on shaping the rhizosphere fungal community of ginseng. To summarize, precise NPK application was an important factor in regulating the structure of fungal communities in ginseng rhizosphere soil, and combined NPK also provided a good microenvironment for ginseng growth. Notably, similar effects of NPK combined application on crop rhizosphere fungal communities have also been observed in other studies (Cai et al., 2021).

### NPK, Yield, Quality, and Fungal Community Interactions

In this study of interactions between plants and soil nutrients and between soil nutrients and soil microorganisms, the fertilization scheme T7 [(N_3_P_1_K_3_): N fertilizer, 50 g·m^−2^; P fertilizer, 15 g·m^−2^; K fertilizer, 60 g·m^−2^] with low P and high N and K significantly increased ginseng yield and ginsenoside contents. The N_3_P_1_K_3_ (T7) treatment also increased relative abundances of potential beneficial flora and decreased those of potential harmful flora. The important plant nutrient element P is a constituent of key molecules, such as nucleic acid and ATP, and participates in physiological processes, such as energy metabolism and photosynthesis (Dissanayaka et al., 2020). Phosphorus limitation leads to strong competition between plants and microorganisms for unstable P resources (Kwatcho et al., 2022). Low P availability suggested that P was the key factor to increase the growth of ginseng fibrous roots and improve root biomass. The addition of N as a necessary element for plant growth and development is an important guarantee to increase income and yield in agricultural production (Khan et al., 2022). At high levels of N, direct effects of N nutrients or indirect changes in soil and plant properties may affect ginseng soil fungal communities. Nitrogen fertilization causes notable changes in N-cycling microbes, especially those in fungal communities (Guo et al., 2018; Du et al., 2019). Potassium is important in carbohydrate and protein metabolism and is recognized as an element that affects the quality (Li et al., 2021b). The application of K fertilizer can increase crop yield and quality, and it is also widely used in preventing and treating plant and soil diseases (Pradip et al., 2016). Those effects could help explain the effect of high K on soil fungi of ginseng roots. The soil used in this study was dark brown soil from the ginseng daodi-producing area and was characterized by weak acidity, high organic matter, and low P and high N and K contents, which were characteristics consistent with the optimal NPK fertilizer combination in this study. In agricultural production, owing to differences in soil environments, textures, and NPK residues, fertilization schemes need to be adjusted and optimized to achieve high ginseng yields and quality.

**Figure 8:**
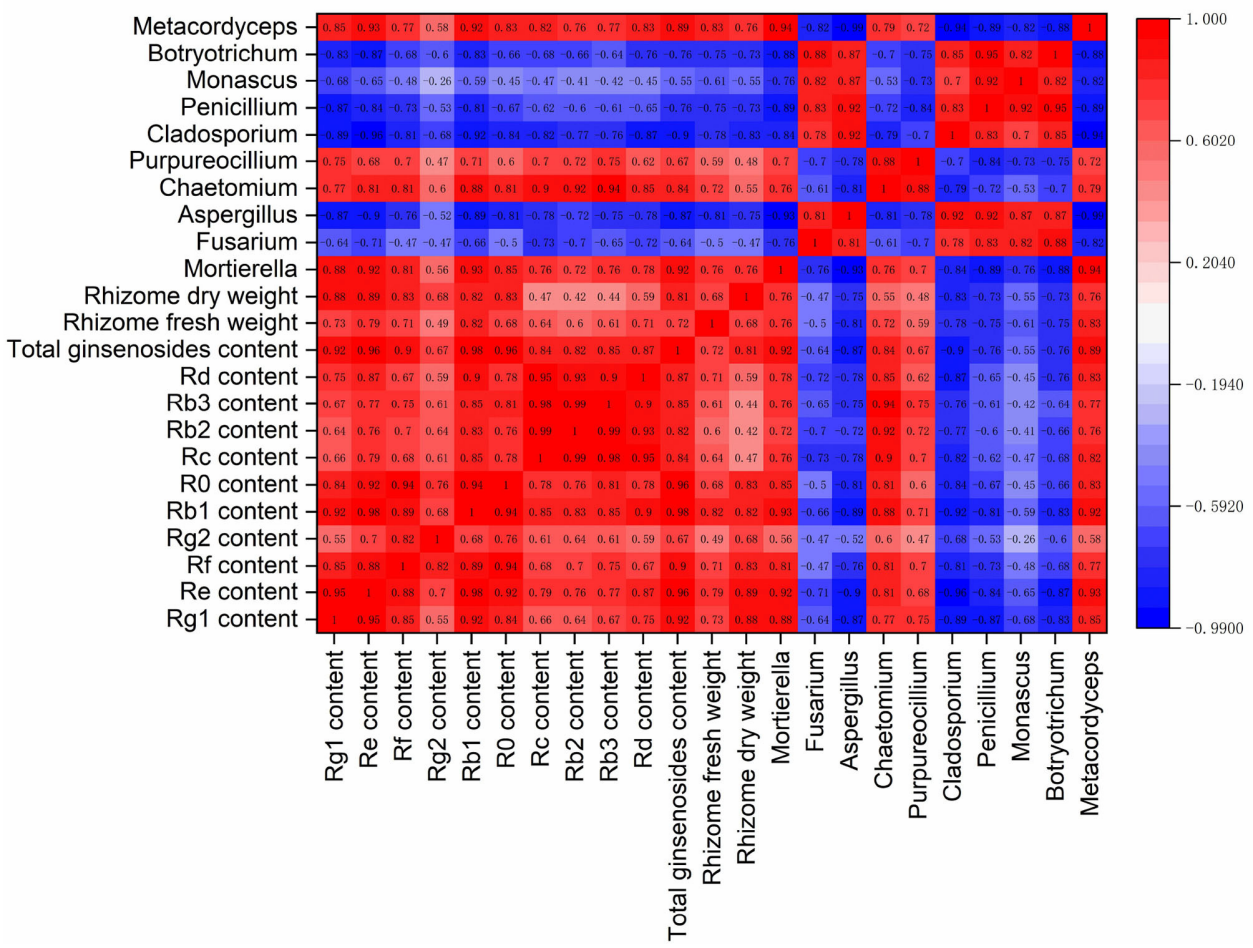
Correlation matrix of ginseng yield, ginsenoside contents, and dominant genera of rhizosphere fungi (*n* = 10).

Notably, there were also relatively high correlations between plants and soil fungi (Figure 8). In this study, soil fungal communities affected ginseng yield, as well as ginsenoside biosynthesis. Relative abundances of *Mortierella, Chaetomium, Purpureocillium*, and *Metacordyceps* were significantly positively correlated with ginseng rhizome yield and ginsenoside contents. The results indicated that the four genera stimulated the production of primary and secondary metabolites in ginseng rhizomes. Similarly, in *Panax notoginseng*, potential beneficial fungi increased the accumulation of saponins (Zhang et al., 2020). Effects of fungal communities on plant metabolism involve dynamic and complex biological processes. For example, rhizosphere fungi affect the accumulation of plant metabolites by regulating the expression of immune system-related genes and the activity of metabolic enzymes (Martínez-Castro et al., 2018; Begum et al., 2020).

*Fusarium* is a common pathogen that in high abundance can infect plants and cause root rot and other diseases (Peng et al., 2022). In this study, potential harmful fungi represented by *Fusarium* were highly negatively correlated with root biomass and ginsenoside accumulation. Plant–soil fungal interactions should not be considered simply as those with beneficial or harmful fungi. Rather, whether such interactions increase plant adaptability to the environment under specific environmental conditions should be considered (Trivedi et al., 2020). In this study, soil fungi were hypothesized to increase NPK transformations and transport of nutrients (Hu et al., 2020; Ning et al., 2020; Lang et al., 2021) and thereby increase nutrient availability for ginseng growth and also protect ginseng from pathogens by competition, production of antibacterial compounds, and hydrolysis (Naik et al., 2019; Delgado-Ospina et al., 2021).

Overall, exogenous NPK nutrients interacted synergistically with soil fungi in the short term and increased the accumulation of ginseng biomass and secondary metabolites. These results provide a theoretical basis to develop efficient cultivation of ginseng. In future studies, the following should be considered: (i) soil bacterial communities also have important effects on crop growth (Shen et al., 2022), and how they affect ginseng cultivation should be investigated; (ii) mechanisms by which soil fungi stimulate the production of secondary metabolites in ginseng rhizomes need further investigation; and (iii) whether precise NPK combined application can increase long-term benefits and also alleviate problems caused by mineral fertilizers on soil fungi and soil functions needs further in-depth and systematic research.

## Conclusion

In this study, the effects of soil nutrients (NPK) on ginseng growth indexes (plant height, stem, and leaf fresh weight, rhizome fresh and dry weights), active compounds (ginsenosides Rg1, Re, Rf, Rg2, Rb1, Ro, Rc, Rb2, Rb3, and Rd), and rhizosphere fungal communities were investigated with the goal to optimize an NPK fertilization program. The N_3_P_1_K_3_ (T7) fertilizer treatment (N fertilizer, 50 g·m^−2^; P fertilizer, 15 g·m^−2^; K fertilizer, 60 g·m^−2^) significantly increased ginseng yield and ginsenoside contents. The N_3_P_1_K_3_ (T7) treatment also affected the structure of the ginseng rhizosphere fungal community and provided a favorable environment for ginseng growth by increasing the relative abundances of potential beneficial fungi and decreasing those of potentially pathogenic fungi. In conclusion, the combined effects of biotic and abiotic processes in agricultural production determine crop yields and quality. Precise NPK fertilization can affect both biotic and abiotic processes and directly increase crop yields and quality, as well as increase crop adaptability to the environment by shaping specific microbial communities.

## Data Availability Statement

The datasets presented in this study can be found in online repositories. The names of the repository/repositories and accession number(s) can be found below: https://www.ncbi.nlm.nih.gov/bioproject/828555.

## Author Contributions

JS: data curation, formal analysis, software, and writing—original draft. HL: methodology. QY, BK, and YJ: sample collection. LW: conceptualization, funding acquisition, and supervision. CX: conceptualization, project administration, funding acquisition, and writing—review and editing. All authors contributed to the article and approved the submitted version.

## Funding

This study was supported by the National Nature Science Foundation of China [81803649].

## Conflict of Interest

The authors declare that the research was conducted in the absence of any commercial or financial relationships that could be construed as a potential conflict of interest.

## Publisher's Note

All claims expressed in this article are solely those of the authors and do not necessarily represent those of their affiliated organizations, or those of the publisher, the editors and the reviewers. Any product that may be evaluated in this article, or claim that may be made by its manufacturer, is not guaranteed or endorsed by the publisher.
