# Supplementary material for: Optimal NPK Fertilizer Combination Increases Panax ginseng Yield and Quality and Affects Diversity and Structure of Rhizosphere Fungal Communities
Source: Front Microbiol. 2022 Jun 21;13:919434. doi: 10.3389/fmicb.2022.919434 (PMC9255912; doi:10.3389/fmicb.2022.919434)
Supplement: Supplementary file 1 [file Data_Sheet_1.docx]

Supplementary Material

# 1 Supplementary Figures and Tables

## 1.1 Supplementary Figures





**Supplementary Figure 1.** Effects of NPK combined application on concentrations of ginsenosides Rg1, Re, Rf, Rg2, Rb1, Ro, Rc, Rb2, Rb3, and Rd in ginseng rhizomes (*n* = 3). Note: (a) Different letters indicate significant differences among treatments at *p* < 0.05, according to SNK tests. (b) Composition of fertilization treatments: CK, N_0_P_0_K_0_; T1, N_1_P_1_K_1_; T2, N_1_P_2_K_2_; T3, N_1_P_3_K_3_; T4, N_2_P_1_K_2_; T5, N_2_P_2_K_3_; T6, N_2_P_3_K_1_; T7, N_3_P_1_K_3_; T8, N_3_P_2_K_1_; T9, N_3_P_3_K_2_.

**
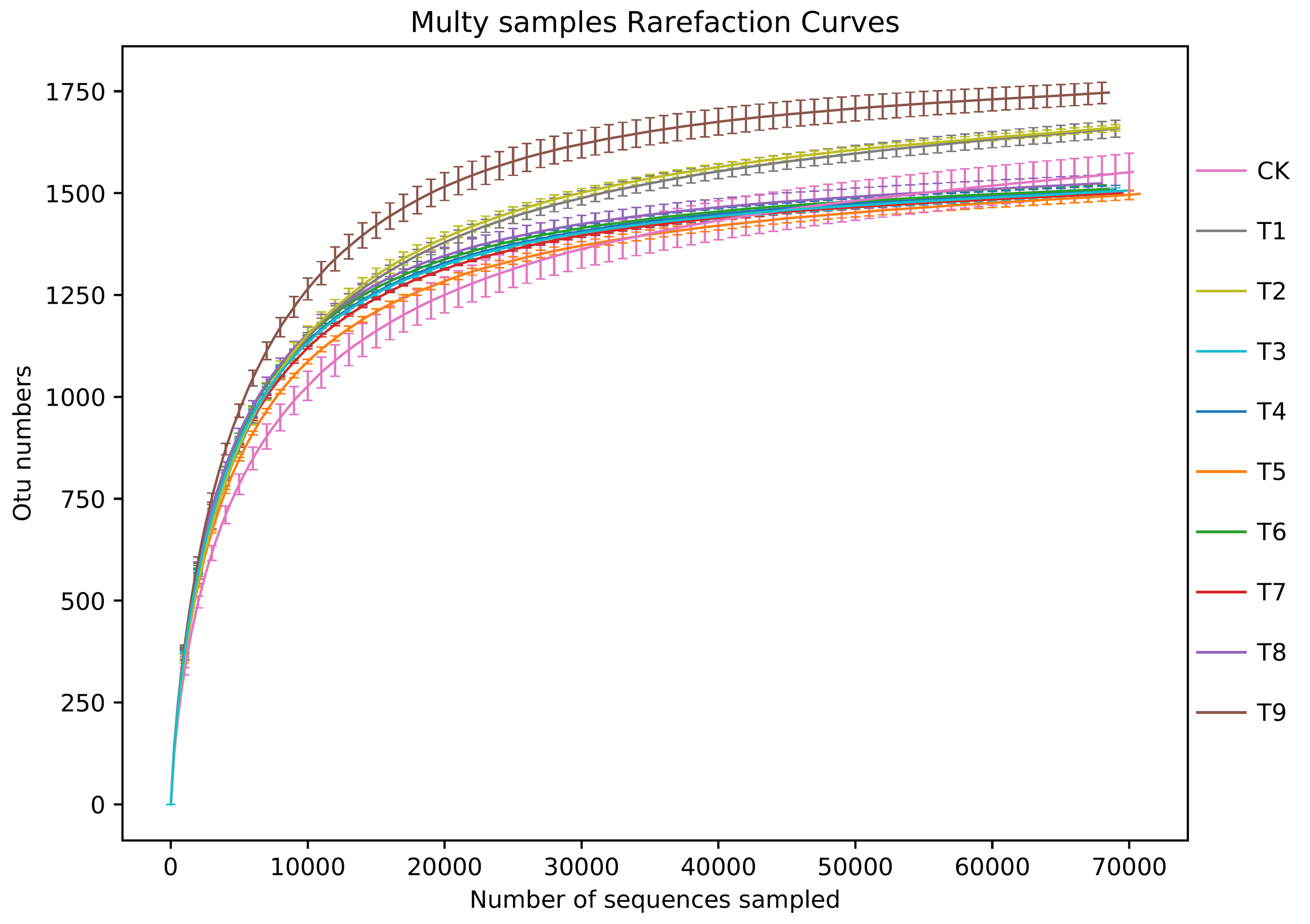
**

**Supplementary Figure 2.** Rarefaction curves for all of the soil samples. Note: (a) Composition of fertilization treatments: CK, N_0_P_0_K_0_; T1, N_1_P_1_K_1_; T2, N_1_P_2_K_2_; T3, N_1_P_3_K_3_; T4, N_2_P_1_K_2_; T5, N_2_P_2_K_3_; T6, N_2_P_3_K_1_; T7, N_3_P_1_K_3_; T8, N_3_P_2_K_1_; T9, N_3_P_3_K_2_.

**1.2 Supplementary Tables**

| **Supplementary Table S1.** Relative ratio of individual saponins to all ten saponins under different fertilization treatments. | | | | | | | | | | |
| --- | --- | --- | --- | --- | --- | --- | --- | --- | --- | --- |
| Treatment | Rg1% | Re% | Rf% | Rg2% | Rb1% | Ro% | Rc% | Rb2% | Rb3% | Rd% |
| CK | 13 | 23 | 5 | 2 | 21 | 16 | 7 | 6 | 1 | 6 |
| T1 | 11 | 18 | 4 | 2 | 24 | 14 | 10 | 9 | 1 | 9 |
| T2 | 12 | 20 | 4 | 3 | 23 | 13 | 9 | 9 | 1 | 6 |
| T3 | 15 | 18 | 5 | 2 | 24 | 16 | 7 | 7 | 1 | 5 |
| T4 | 9 | 16 | 3 | 2 | 26 | 16 | 10 | 11 | 1 | 6 |
| T5 | 8 | 17 | 4 | 3 | 22 | 12 | 11 | 14 | 2 | 7 |
| T6 | 8 | 19 | 3 | 3 | 22 | 13 | 11 | 13 | 2 | 6 |
| T7 | 11 | 19 | 5 | 3 | 24 | 16 | 8 | 10 | 1 | 5 |
| T8 | 10 | 21 | 4 | 3 | 22 | 11 | 10 | 10 | 1 | 7 |
| T9 | 9 | 21 | 4 | 4 | 24 | 16 | 8 | 8 | 1 | 5 |

Note: (a) Values are mean (n = 3). (b) Composition of fertilization treatments: CK, N_0_P_0_K_0_; T1, N_1_P_1_K_1_; T2, N_1_P_2_K_2_; T3, N_1_P_3_K_3_; T4, N_2_P_1_K_2_; T5, N_2_P_2_K_3_; T6, N_2_P_3_K_1_; T7, N_3_P_1_K_3_; T8, N_3_P_2_K_1_; T9, N_3_P_3_K_2_.

| **Supplementary Table S2.** Relative abundance ( % ) of fungal phyla in all treatments. | | | | | | | | | | |
| --- | --- | --- | --- | --- | --- | --- | --- | --- | --- | --- |
| phylum | CK | T1 | T2 | T3 | T4 | T5 | T6 | T7 | T8 | T9 |
| Ascomycota | 79.25 | 72.98 | 74.23 | 62.09 | 62.51 | 68.28 | 63.59 | 61.99 | 61.93 | 69.19 |
| Mortierellomycota | 6.30 | 8.72 | 8.29 | 15.81 | 15.75 | 13.19 | 14.52 | 16.33 | 14.14 | 10.86 |
| Basidiomycota | 9.04 | 11.73 | 11.06 | 10.67 | 11.19 | 8.94 | 10.57 | 10.65 | 11.61 | 11.51 |
| Glomeromycota | 0.62 | 0.94 | 1.03 | 3.80 | 3.38 | 4.00 | 3.29 | 4.06 | 3.47 | 2.29 |
| Chytridiomycota | 0.35 | 1.45 | 1.01 | 1.37 | 1.44 | 0.50 | 1.47 | 1.17 | 1.71 | 1.29 |
| Rozellomycota | 1.17 | 0.88 | 0.98 | 0.64 | 0.49 | 0.74 | 0.63 | 0.60 | 0.70 | 0.82 |
| Olpidiomycota | 0.34 | 0.22 | 0.25 | 0.35 | 0.30 | 0.31 | 0.38 | 0.38 | 0.33 | 0.30 |
| Mucoromycota | 0.01 | 0.03 | 0.05 | 0.19 | 0.18 | 0.05 | 0.14 | 0.15 | 0.16 | 0.07 |
| Zoopagomycota | 0.11 | 0.07 | 0.10 | 0.02 | 0.05 | 0.05 | 0.04 | 0.03 | 0.04 | 0.08 |
| Basidiobolomycota | 0.02 | 0.07 | 0.05 | 0.00 | 0.00 | 0.00 | 0.01 | 0.00 | 0.00 | 0.05 |
| Others | 0.01 | 0.03 | 0.03 | 0.02 | 0.02 | 0.01 | 0.02 | 0.01 | 0.03 | 0.03 |
| Unclassified | 2.78 | 2.87 | 2.93 | 5.03 | 4.67 | 3.94 | 5.34 | 4.62 | 5.89 | 3.52 |

Note: (a) Values are mean (n = 3). (b) Composition of fertilization treatments: CK, N_0_P_0_K_0_; T1, N_1_P_1_K_1_; T2, N_1_P_2_K_2_; T3, N_1_P_3_K_3_; T4, N_2_P_1_K_2_; T5, N_2_P_2_K_3_; T6, N_2_P_3_K_1_; T7, N_3_P_1_K_3_; T8, N_3_P_2_K_1_; T9, N_3_P_3_K_2_.

| **Supplementary Table S3.** Relative abundance (%) of fungal genera in all the treatments. | | | | | | | | | | |
| --- | --- | --- | --- | --- | --- | --- | --- | --- | --- | --- |
| genus | CK | T1 | T2 | T3 | T4 | T5 | T6 | T7 | T8 | T9 |
| *Mortierella* | 5.42 | 8.21 | 7.54 | 14.89 | 14.85 | 12.14 | 13.59 | 15.34 | 13.24 | 10.16 |
| *Fusarium* | 9.30 | 8.42 | 7.64 | 6.01 | 5.71 | 6.68 | 5.46 | 5.80 | 5.21 | 6.87 |
| *Aspergillus* | 6.97 | 5.43 | 5.11 | 3.00 | 2.95 | 3.92 | 3.84 | 3.02 | 3.21 | 4.53 |
| *Chaetomium* | 1.74 | 1.82 | 1.80 | 5.66 | 5.73 | 6.23 | 4.95 | 5.53 | 5.08 | 3.36 |
| *Purpureocillium* | 0.67 | 0.80 | 0.83 | 3.65 | 3.32 | 3.62 | 3.22 | 3.09 | 3.57 | 1.69 |
| *Cladosporium* | 2.36 | 2.60 | 2.43 | 1.59 | 1.48 | 1.66 | 2.00 | 1.53 | 1.59 | 2.26 |
| *Penicillium* | 3.43 | 3.00 | 2.22 | 0.97 | 1.10 | 1.28 | 1.19 | 1.05 | 1.00 | 1.96 |
| *Monascus* | 2.38 | 1.95 | 1.54 | 1.25 | 1.29 | 1.71 | 1.32 | 1.48 | 1.28 | 1.54 |
| *Botryotrichum* | 4.61 | 2.15 | 3.09 | 0.62 | 0.65 | 0.75 | 0.70 | 0.62 | 0.60 | 1.78 |
| *Metacordyceps* | 0.20 | 0.30 | 0.35 | 2.00 | 2.22 | 1.85 | 1.91 | 2.18 | 1.92 | 1.00 |
| Others | 40.54 | 45.08 | 46.19 | 38.30 | 38.91 | 40.25 | 38.83 | 38.65 | 39.30 | 43.54 |
| Unclassified | 22.37 | 20.23 | 21.28 | 22.05 | 21.80 | 19.93 | 23.00 | 21.71 | 24.02 | 21.31 |

Note: (a) Values are mean (n = 3). (b) Composition of fertilization treatments: CK, N_0_P_0_K_0_; T1, N_1_P_1_K_1_; T2, N_1_P_2_K_2_; T3, N_1_P_3_K_3_; T4, N_2_P_1_K_2_; T5, N_2_P_2_K_3_; T6, N_2_P_3_K_1_; T7, N_3_P_1_K_3_; T8, N_3_P_2_K_1_; T9, N_3_P_3_K_2_.
